# Large Rectal Neuroendocrine Tumor Curatively Resected Using Loop Ligation Device

**DOI:** 10.14309/crj.0000000000002241

**Published:** 2026-07-20

**Authors:** Lauren Matlack, Sherifatu Abu, Shruti Sirapu, Juhi Mittal, Zachary Wilmer Reichenbach, Malorie Simons, Nicholas McDonald

**Affiliations:** 1 Department of Gastroenterology and Hepatology, Temple University Hospital, Philadelphia, Pennsylvania; 2 Department of Medical Oncology, Temple University Hospital, Philadelphia, Pennsylvania; 3 Center for Substance Abuse Research (CSAR), Lewis Katz School of Medicine, Temple University, Philadelphia, Pennsylvania; 4 Center for Microbiology and Immunology, Lewis Katz School of Medicine, Temple University, Philadelphia, Pennsylvania; 5 Division of Gastroenterology and Hepatology, Department of Medicine, Fox Chase Cancer Center, Philadelphia, Pennsylvania

**Keywords:** rectum, neuroendocrine tumor, endoscopic mucosal resection, loop ligation

## Abstract

Rectal neuroendocrine tumors (NETs) account for a small portion of all rectal neoplasms, however, encompass approximately 20% of all NETs arising from the gastrointestinal tract or pancreas. Band ligation with hot en bloc endoscopic mucosal resection (EMR) is highly effective and frequently used for resection of smaller NETs. We present a case of a NET that was unable to be resected by standard EMR and endoscopic full-thickness resection due to size, or endoscopic submucosal dissection given complexity, time, cost, and complication rates, and was resected using underwater EMR technique with a loop ligation device and hot snare.

## INTRODUCTION

Rectal neuroendocrine tumors (NETs) account for 1%–2% of all rectal neoplasms and 12%–27% of all NETs arising from the gastrointestinal tract or pancreas.^1^ The incidence rate of rectal NET has risen due to colorectal cancer screening and improved detection of subepithelial lesions.^1^ Most rectal NETs are small, well-differentiated, and confined to the submucosa (layer 3), allowing for curative endoscopic resection and complete R0 resection.^2^

Band ligation endoscopic mucosal resection (EMR) is commonly used for rectal NETs ≤10 mm and is associated with high rates of en bloc and complete resection.^3,4^ However, lesion size, morphology, and inability of the lesion to fit within the distal attachment cap can limit the feasibility of band ligation EMR or endoscopic full thickness resection (EFTR). Endoscopic submucosal dissection (ESD) is an effective alternative as it provides high curative resection rates but is technically demanding, time-intensive, and not universally available.^5^ We present a case of 2 concurrent rectal NETs, including a lesion that was not amenable to standard band ligation EMR or EFTR due to size but was curatively resected using underwater EMR technique with a loop ligation device, and hot snare.

## CASE REPORT

A 55-year-old woman with a history of pancreatic ductal adenocarcinoma status post distal pancreatectomy, and previously diagnosed NETs, presented for follow-up of her known rectal NETs.

Three years prior, the patient had undergone screening colonoscopy which identified 2 rectal subepithelial lesions measuring 12 mm and 20 mm, respectively. Biopsy of the larger lesion demonstrated a grade I, well-differentiated rectal NET. Endoscopic resection was deferred at that time as the patient was subsequently diagnosed with pancreatic ductal adenocarcinoma and was lost to follow-up.

The patient returned to the clinic to reassess the rectal lesions and pursue definitive endoscopic resection. Colonoscopy revealed 2 yellow, subepithelial lesions in the rectum consistent with known NETs, which were identified 8 cm from the anal verge by endoscopic ultrasound. Both appeared to be hypoechoic, nodular, and arising from the submucosa (layer 3) by endoscopic ultrasound.

**Figure 1.**
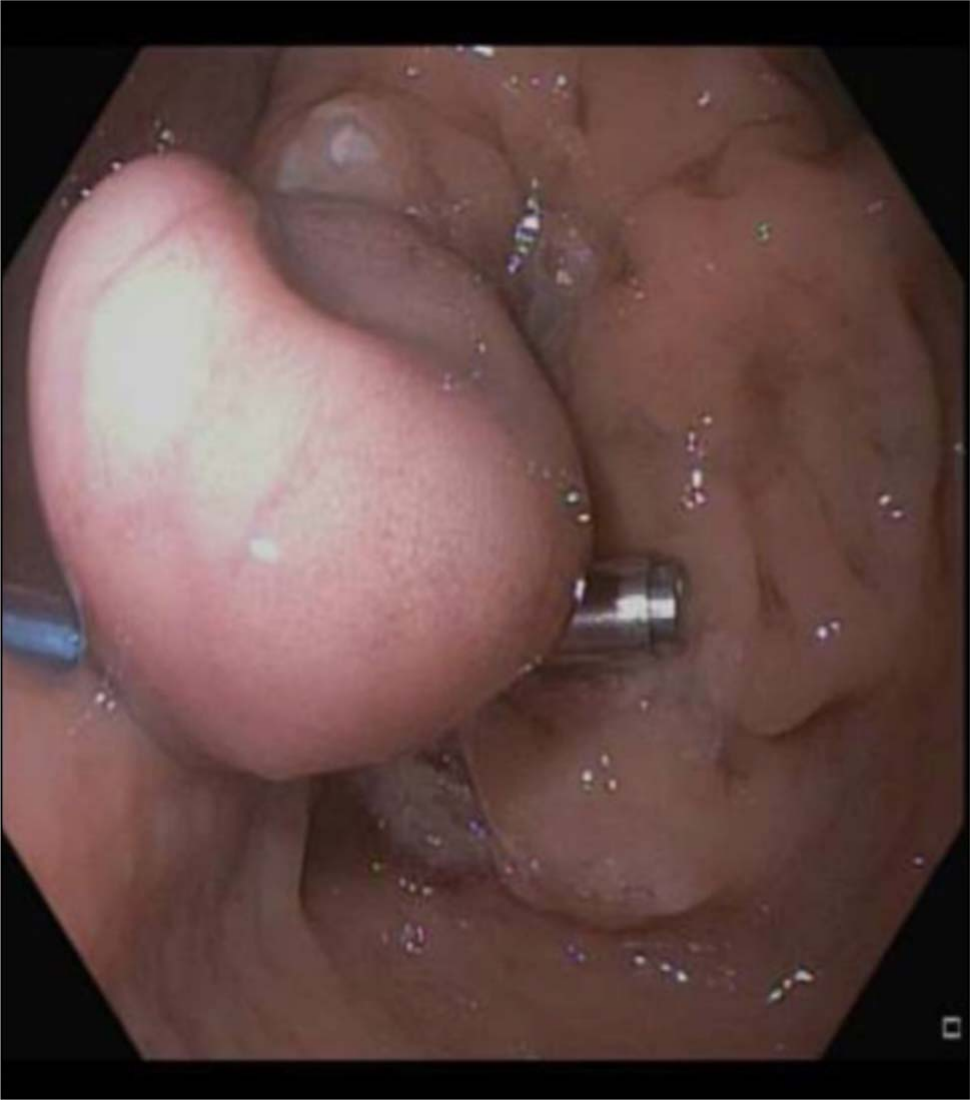
20 mm neuroendocrine tumor located 8 cm from the anal verge with loop ligation device placed around the base of the lesion.

**Figure 2.**
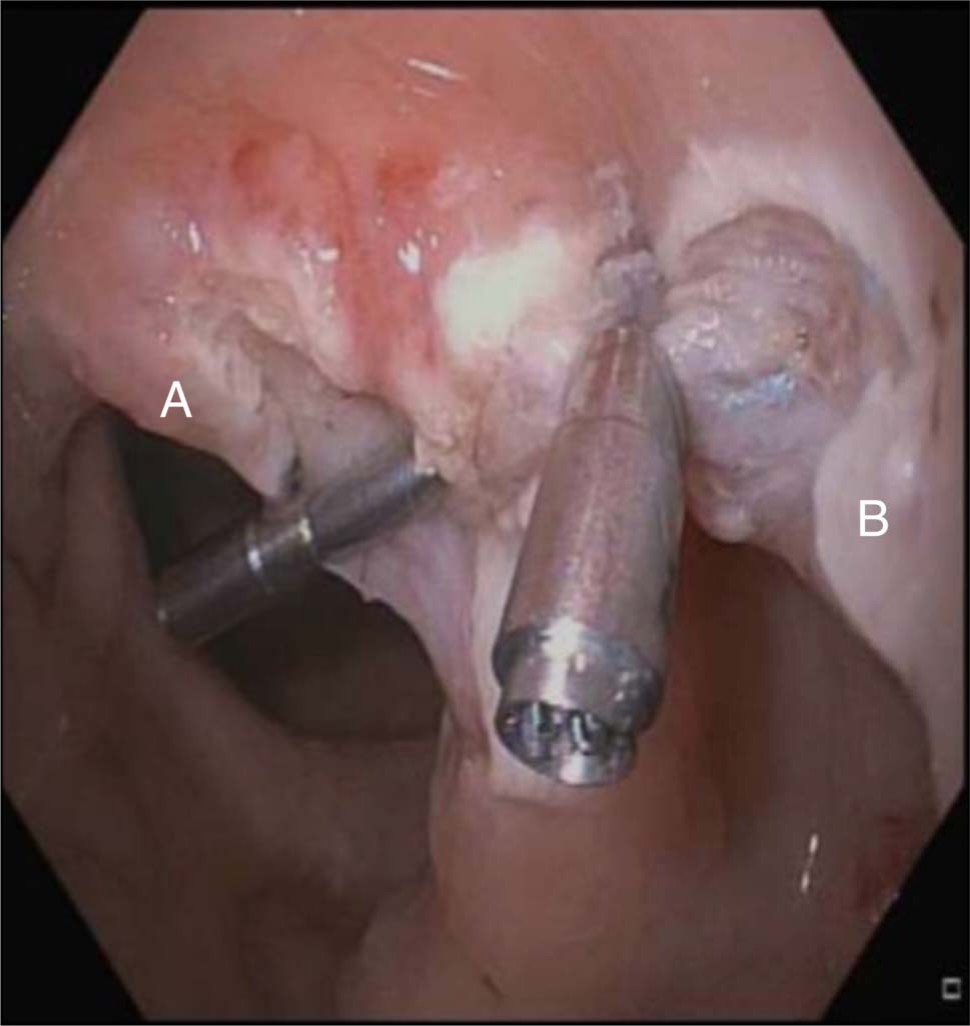
The smaller lesion (A) and the larger lesion (B) sites after endoscopic mucosal resection and closed with through-the-scope clips.

The smaller lesion was successfully resected en bloc using conventional band ligation EMR and the defect was closed with through-the-scope clips. Based upon the larger lesion's diameter and morphology, the circumference of the lesion did not fit in the distal cap for band ligation EMR or EFTR. As such, an endoloop ligation device was used to grasp the lesion and surrounding tissue by being placed at the base of the lesion (Figure 1) to achieve a banding effect followed by hot snare en bloc resection using underwater technique which eliminated the need for submucosal injection and created a floating effect to lift the lesion away from the muscularis propria (Figure 2). One through-the-scope clip was placed to close the defect. No immediate complications were identified following the procedure after careful inspection.

**Figure 3.**
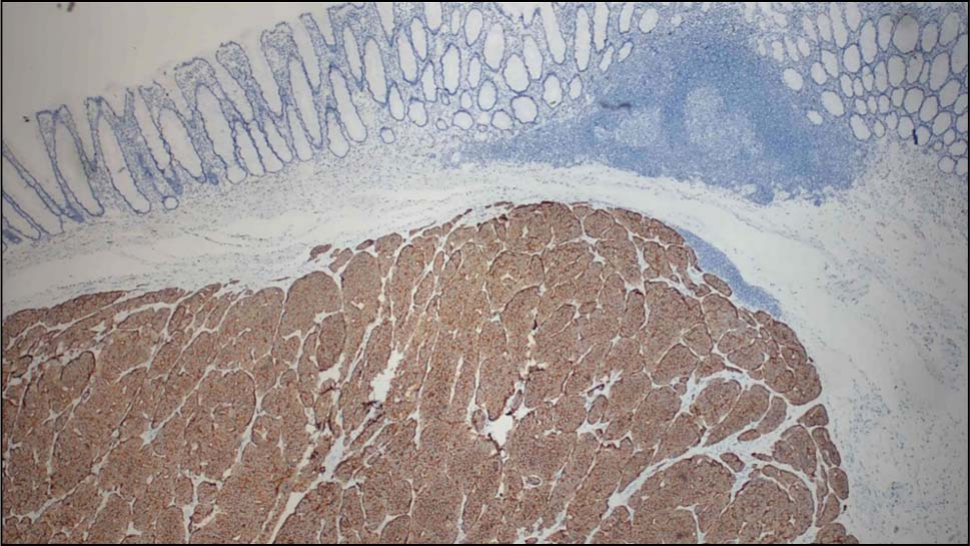
Synaptophysin stain, viewed on 4× power, of the specimen obtained by endoloop ligation technique that resulted in grade II, well-differentiated rectal neuroendocrine tumor.

Histopathologic evaluation of both lesions returned as grade II, well-differentiated rectal NETs with negative deep and lateral margins, consistent with R0 (curative) resection (Figure 3) and extent of invasion to the submucosa. Ki67 was 4% positive in the smaller lesion and 5% positive in the larger lesion. The patient was followed in the outpatient clinic and did not experience any postprocedure complications. Based on the National Comprehensive Cancer Network guidelines, this patient with a rectal NET resection 20 mm or less, with negative margins, will undergo routine surveillance with endoscopy annually for 5 years.

## DISCUSSION

According to Surveillance Epidemiology and Ends Results database, rectal NETs make up approximately 16% of all neuroendocrine tumors and have the best prognosis compared with other NETs.^6^ Endoscopic resection is the preferred treatment of small (<10 mm), localized rectal NETs without high-risk features as it offers excellent oncologic outcomes. Currently, the American College of Gastroenterology does not have a preferred endoscopic ligation technique for the resection of small (<10 mm), low-grade NETs.^7^ However, band ligation EMR is widely favored for lesions ≤10 mm due to its technical simplicity and high rates of curative resection.^8,9^ In this case, surgical resection could have been considered given the large size, however the lesion was able to be resected endoscopically using the band ligation device. The overall size of the lesion was too large to fit into an EFTR cap, and it was felt that the lesion could be resected en bloc with loop ligation device and snare with a decreased procedure length, cost, and suspected R0 resection. In addition, patient convenience was considered as the patient had previously been lost to follow-up, and in this case the patient could undergo resection during 1 session. The low adverse events associated with endoscopic resection compared with surgical resection or ESD were weighed in this case as well.

Endoloop-assisted underwater EMR has previously been described for resection of rectal polyps and gastrointestinal stromal tumors; however, to our knowledge, its use for rectal NETs has not been previously reported. Other endoscopic approaches such as EFTR similarly require lesions to fit in the device cap, limiting its use to tumors ≤20 mm. ESD is often recommended for larger rectal NETs (without high-risk features) confined to the submucosa as it allows en bloc resection regardless of lesion size.^7^ Despite its effectiveness, ESD requires advanced expertise, longer procedure times, and may not be readily available in all practice settings. Despite limited data, ESD can be considered in cases to allow for en bloc resection to obtain large negative margins regardless of the size of the lesion to provide an R0 cure.^10^ The risk of adverse events such as bleeding and perforation are small (<2%) but more likely to occur compared with EMR. In cases of rectal NETs that are larger than 20 mm but still technically feasible for EMR, loop ligation devices can be considered as an alternative method to achieve curative resection. Surgical resection is preferred for lesions >20 mm or 10–20 mm with high-risk features.^8,9^

In this case, an endoloop-assisted underwater EMR technique enabled successful en bloc resection of a rectal NET 20 mm in diameter. The endoloop ligation provided mechanical compression and delineation of the lesion while underwater EMR increased lesion mobility and facilitated snare capture. This hybrid approach allowed curative (R0) resection without the need for surgery or ESD and thus represents a practical alternative for rectal NETs confined to submucosa and too large for conventional band ligation EMR. This case demonstrates loop ligation as a viable alternative for larger NETs, wherein ESD may be more time consuming, expensive, and have a higher complication rate, and EFTR is not an option owing to the large size of the lesion.

## DISCLOSURES

Author contributions: L. Matlack, S. Abu, and N. McDonald wrote the manuscript. N. McDonald performed the procedures. All authors performed critical review and editing of the manuscript. N. McDonald is the article guarantor.

Financial disclosure: None to report.

Informed consent was obtained for this case report.

## ABBREVIATIONS:

EFTR: endoscopic full thickness resection
EMR: endoscopic mucosal resection
ESD: endoscopic submucosal dissection
NETs: neuroendocrine tumors
